# Examining the Structure of Negative Affect Regulation and Its Association With Hedonic and Psychological Wellbeing

**DOI:** 10.3389/fpsyg.2018.01592

**Published:** 2018-08-29

**Authors:** Alicia Puente-Martínez, Darío Páez, Silvia Ubillos-Landa, Silvia Da Costa-Dutra

**Affiliations:** ^1^Department of Social Psychology and Methodology of Behavioural Sciences, University of Basque Country, Gipuzkoa, Spain; ^2^Department of Social Psychology, Faculty of Health Sciences, University of Burgos, Burgos, Spain

**Keywords:** measure of affect regulation, hedonic and psychological well-being, coping, anger, sadness

## Abstract

The present study examines the structure of negative affect regulation strategies by confirmatory factor analysis. A total of 264 students (*n* = 187 women, 65 men) (*M* = 24 years; *SD* = 9.32) took part in this study. Results show a good fit indices for a three facets model: (1) modification of situation (problem-directed action, seeking emotional and instrumental social support, psychological abandonment and social isolation); (2) attentional deployment and cognitive change (distraction, acceptance, gratitude, rumination, reappraisal, spirituality, and social comparison); and (3) response modification (suppression, active and passive physiological, humor and warmth, venting, confrontation, and regulated emotional expression). The scale validity is confirmed through correlations between the expanded of Mood Affect Regulation Scale dimensions including dimensions of dispositional reappraisal and suppression, and hedonic and psychological well-being. Participants report an adaptive profile with high psychological well-being, even if they report low positive affect, suggesting a greater relevance of eudaimonic than hedonic well-being for affect regulation.

## Introduction

The way positive and negative emotions are regulated can have a crucial impact on our well-being (Bryant and Veroff, 2007; Gross, 2015). There are various theories on self-regulation; however, there is consensus that it includes skills or conducts such as planning; cognitive, and meta-cognitive aspects such as self-tracking and motivational aspects such as setting goals. Nevertheless, there are few attempts to contrast the whole structure of a large repertoire of forms of self-regulation to the management of negative affect (Augustine and Hemenover, 2008; Webb et al., 2012; Peña-Sarrionandia et al., 2015; Naragon-Gainey et al., 2017).

Gross et al. (2006) only examined reappraisal and suppression of emotions trough the Emotional Regulation Questionnaire (ERQ) scale. Prizmic and Larsen (2012) described around 20 coping and affect regulation strategies with the 32 item Mood Affect Regulation Scale (MARS) (Barber et al., 2010). However, the psychometric characteristics of the MARS questionnaire have not yet been studied. In the Spanish adaptation, a similar number of coping and affect regulation forms have been applied using an expanded 56 item version of the MARS scale, indicating that a nucleus of regulation was associated with the attainment of adaptive goals (Páez et al., 2012, 2013; Naragon-Gainey et al., 2017). In fact, different studies have shown a relationship between hedonic and eudaimonic well-being and affect regulation (Gross, 2015). For instance, “flourishing people” reporting simultaneous high hedonic and eudaimonic well-being, use less suppression as a form of emotional regulation (Barber et al., 2010).

Hedonic well-being is typically referred to as being composed of two different elements, emotional well-being (positive/negative affect) and cognitive well-being or life satisfaction (De Leersnyder et al., 2013). Regulation of affect is related to hedonic goals like increasing positive emotions and decreasing negative ones (Larsen and Prizmic, 2008). Functional strategies should be related to more positive rather than negative emotions (Fredrickson, 2009). Psychological or eudaimonic well-being concerns human potential and a satisfactory global mental health, including purpose in life, autonomy, personal growth, self-esteem or acceptance, mastery and positive relations with others (Keyes et al., 2002). Also, psychological well-being is related to emotional and affect regulation (Korpela et al., 2018) because this process implies goals such as increasing self-esteem and showing a positive self-image, situation facing and control increase, and social integration or relatedness (Koole, 2009). That is to say, affect strategies should be related to psychological well-being, through dimensions associated with previous goals, like self-acceptance, mastery or control, and positive relations with others (Ryff, 1989).

Following the taxonomy of regulation proposed by Gross and John (2003), strategies of affect regulation have been divided into (1) modification of situation, (2) attentional deployment and cognitive change, and (3) emotional response modulation.

Modification of situation is a psychological process similar to a functional problem-focused coping strategy and improves negative affect (Skinner et al., 2003). It was found that problem-directed action and planning how to avoid problems is associated with low negative and high positive affect (Larsen and Prizmic, 2008). An integration of five meta-analyses (Penley et al., 2002; Campos M. et al., 2004; Augustine and Hemenover, 2008; Aldao et al., 2010; Webb et al., 2012) about coping, affect regulation and emotional wellbeing estimated the association between affect regulation strategies and emotional balance, and they showed a correlation between direct coping and high wellbeing and positive affectivity, *r* = 0.23 (Páez and Da Costa, 2014). Modification of situation is also associated with emotional intelligence (EI) (*r* = 0.42) (Peña-Sarrionandia et al., 2015). Seeking social support is adaptive for negative affect, particularly when coupled with instrumental responses (Skinner et al., 2003). Finally, helping others and altruistic or pro-social behaviors are a form of functional regulation for negative affect (Skinner et al., 2003). Withdrawing from the situation, or psychological abandonment, is usually related to dysfunctional outcomes. Previous meta-analyses found negative associations between psychological abandonment and affect balance, *r* = -0.28 (Páez and Da Costa, 2014) and with EI, *r* = -0.24 (Peña-Sarrionandia et al., 2015). Individuals with a flourishing state of well-being use fewer avoidance strategies as a form of emotional regulation (Barber et al., 2010). Social isolation is another avoidant dysfunctional form of emotional regulation (Larsen and Prizmic, 2008). Furthermore, there is a negative association between social isolation and affect balance, *r* = -0.31 (Páez and Da Costa, 2014).

Affect regulation could seek to change how a person perceives an emotional situation (Gross, 2015). Distraction or removing oneself cognitively and behaviorally from the negative emotional episode is a functional attentional strategy (Augustine and Hemenover, 2008). Distraction activities that are rewarding and that involve some degree of activity produce positive emotions (Larsen and Prizmic, 2008). Thus, Páez and Da Costa (2014) find a positive association between distraction and positive affect (*r* = 0.17) and Peña-Sarrionandia et al. (2015) between distraction and IE (*r* = 0.17). Strategies such as acceptance or accepting the reality of the event are also adaptive (Aldao et al., 2010; Naragon-Gainey et al., 2017). Also, focusing attention on positive aspects of life and feeling gratitude and self-reward for them is associated with well-being (Larsen and Prizmic, 2008). An integration of meta-analysis found a positive association between acceptance and affect balance, *r* = 0.30 (Páez and Da Costa, 2014), and also with EI, *r* = 0.30 (Peña-Sarrionandia et al., 2015). In contrast, rumination, or repetitive thinking on the causes and consequences of emotions, intensifies emotion in general and is linked to negative affect (Aldao et al., 2010), affect balance, *r* = -0.30 (Páez and Da Costa, 2014) and IE, *r* = -0.17 (Peña-Sarrionandia et al., 2015). However, one meta-analysis found a positive effect of rumination on wellbeing (Augustine and Hemenover, 2008), probably because it was connected to emotional processing, and trying to understand and analyze feelings. Individuals may regulate emotions and affect also by cognitive change. A form of functional cognitive change is a positive reappraisal or perceiving the positive aspects of events and behaviors or distancing from the situation (Larsen and Prizmic, 2008; Jamieson et al., 2013). Research has confirmed that reappraisal is associated with low negative affect and also with positive affect (Nezlek and Kuppens, 2008), with high psychological wellbeing (Barber et al., 2010), with a positive affect balance, *r* = 0.17 (da Costa et al., 2014) and with EI, *r* = 0.18 (Peña-Sarrionandia et al., 2015). Seeking meaning through religion is also an instance of positive reappraisal, related to low negative affect and also to positive affect (Skinner et al., 2003). Social comparison is another cognitive process associated with emotion regulation (Rimé, 2009). Downward social comparison is assumed to have a positive affective influence in the case of negative-affect loaded episodes, and upward social comparison is expected to have a motivational positive effect. Nevertheless, people who often compare themselves with others are less happy (Fujita, 2008), and high hedonic and psychological well-being is negatively associated with social comparison (Barber et al., 2010).

Response modulation includes modification of physiological, subjective and expressive reactions (Páez et al., 2012). Active physiological regulation by exercise or relaxation decreases negative affect, as well as it improves wellbeing, while passive physiological regulation of emotions by eating, drinking, or sleeping is an avoidant dysfunctional response (Biddle and Ekkekakis, 2006). Activation of positive emotions such as affection and humor are also functional forms of regulation used to control especially negative feelings like anger (Kennedy-Moore and Watson, 1999). Humor, involving laughing at one’s own mistakes or faults and those of others but without scorn, seeking merely to lighten the mood, is associated with psychological wellbeing (Martin, 2007; Koval et al., 2014) and is associated with IE, *r* = 0.34 (Peña-Sarrionandia et al., 2015). Furthermore, the ability to regulate the emotional expressive behavior is associated with greater well-being possibly because it helps people adapt flexibly to situational demands (communicate attitudes, goals, and intentions in an adaptive way) (Côté et al., 2010). Venting or the strong non-verbal and behavioral expression of emotions (Augustine and Hemenover, 2008) is a form of regulation that enhances negative affect (Larsen and Prizmic, 2008) and correlates negatively with EI, *r* = -0.13 (Peña-Sarrionandia et al., 2015). However, Páez and Da Costa (2014) found a non-significant association between venting and affect balance, *r* = 0.04. Confrontation or the expression of emotion to those responsible for the negative emotions, with the aim of changing what happened, is usually dysfunctional or neutral in the case of negative affect (Penley et al., 2002). Also, inhibition of feelings and suppression of expressions are dysfunctional forms of emotional regulation for negative affect (Gross, 2005, 2015; Hu et al., 2014), and are negatively associated with well-being (Gross and John, 2003). Both are related to affect balance, *r* = -0.16 and *r* = -0.18 (Páez and Da Costa, 2014) and suppression is associated with EI, *r* = -0.21 (Peña-Sarrionandia et al., 2015).

Based on the above, the research question of this study is to examine (1) whether emotional regulation strategies are structured in the phases of regulation proposed by Gross (2015), and (2) how they are associated with hedonic and psychological well-being.

### Objectives of the Study

The aim of the present research is to examine (a) the psychometric properties and the structure of regulation of negative affect in the different facets of Gross’ model (2015) and (b) the association with hedonic and eudemonic well-being. While previous literature has usually analyzed dispositional regulation strategies (Barber et al., 2010) applied to abstract negative events, we will explore how individuals apply the same strategies to negative emotional episodes of sadness and anger.

We also expect to find a congruent relationship between the use of functional and dysfunctional strategies in episodes of anger and sadness with dispositional indicators of emotional regulation and hedonic and psychological well-being.

## Materials and Methods

### Participants

The total sample is composed by 264 students (*n* = 187 women and 65 men, and *n* = 12 was missing data) who took part in this study. The students were from three different Spanish universities, and were consulted during different practice sessions (in the 3rd year and in the adult university cases). The mean age was 24 years (*SD* = 9.32, range 18–71 years) where only 10.6% were working and 72.7% studying too.

### Instruments

The instruments applied include an affective regulation scale (MARS), a dispositional regulation criterion variable (ERQ) and two indicators of wellbeing, one related to hedonic (PANAS) and another psychological (PWB) wellbeing. Two simple questions were also included to measure the intensity of negative events (sadness and anger) (1: low intensity and 10: high intensity) and how (un)pleasant emotional experiences were (1: unpleasant- 8: pleasant).

#### Measure of Affect Regulation Styles, MARS (Larsen and Prizmic, 2004; Páez et al., 2012)

The MARS scale is originally made up of 32 items, to which a further 24 items version was added, generated on the basis of the previous emotional regulation scales (Nezlek and Kuppens, 2008; Quoidbach et al., 2010), so as to adequately represent different forms of emotional regulation (see **Table 1**). It refers to the events of the last 12 months with a Likert-type response scale, ranged from 0 (never) to 6 (nearly always). Higher scores indicate greater use of these forms of coping and emotional regulation in negative emotional episodes. The internal consistency for the extended version of the MARS scale was good, with α between 0.61 and 0.91 (Páez et al., 2012) and between 0.52 and 0.92 (Páez et al., 2013). **Table 1** presents the dimensions, the items and the descriptive statistics for each item. The *g* factor exhibited a ω_h_ coefficient of 0.88 the modification of situation dimension (total variance: 0.62, common variance: 0.62); 0.76 cognitive and attentional change (total variance: 0.29, common variance: 0.36), and 0.75 (total variance: 0.29, common variance: 0.35) for the response modulation subscale.

**Table 1:** Descriptive statistics for each item.

Item	Anger and sadness		
	α	*M*	*DT*
**Modification of situation-** *split-half reliability- α _1_ = 0.91, α_2_ = 0.70, r = 0.58 Problem-directed action* (F)	**0.89**		
**04.** I made a plan or resolution to change this situation		5.63	3.18
**05.** I took action to solve the problem causing my mood		6.01	3.16
06. I made a plan or resolution to avoid such problems in the future or to maintain a positive situation		6.17	3.11
*Social support emotional* (F)	**0.92**		
**52**. I talked to someone about my feelings		6.06	3.43
53. I spoke in order to get understanding and support		5.62	3.41
*Instrumental and informative social support* (F)	**0.93**		
54. I talked to someone in order to resolve or improve the situation that triggered my mood		4.64	3.46
**55.** I talked to an advisor or counselor		5.41	3.50
56. I asked someone who had faced a similar problem or situation what they did		4.16	3.38
*Withdrawal* (D)	**0.66**		
**07**. I withdrew from or avoided the situation		2.88	2.42
08. I carried on as if nothing had happened		2.65	2.66
09. I gave up, did nothing; I did not attempt to control the situation		2.17	2.26
**35.** I tried to accept it as my fate: what will be, will be		4.81	3.24
*Social isolation* (D)	**0.52**		
13. I withdrew from or avoided the persons related to the situation		3.52	2.70
**14.** I kept myself to myself, I wanted to be alone		3.61	2.94
*Altruism* (F)			
**41.** I went out of my way to help someone		3.20	2.86
**Attentional deployment and cognitive change-** *split-half reliability- α_1_ = 0.92, α_2_ = 0.70, r = 0.93*			
*Rumination* (D)	**0.84**		
01. I thought about how I could have done things differently		5.29	3
**02.** I tried to understand my feelings by thinking about and analyzing them		6.49	3.08
03. I thought quickly about what had happened, about the emotional effects of the situation		6.49	2.83
*Distraction* (F)	**0.89**		
**21.** I did something fun, something I really enjoy		5.63	2.98
**22.** I watched TV, read a book, etc., for distraction		5.70	2.85
**23.** I worked on something or stayed busy to forget my mood		5.43	2.92
**24.** I thought about something to distract myself from my feelings		5.33	2.72
**25.** I socialized to forget my mood		5.93	2.84
*Wishful thinking* (D)			
**29.** I daydreamed about the time when I will feel better than today		4.91	3.08
*Acceptance and self-control* (F)	**0.69**		
32. I counted to 10 before answering, in an effort to avoid overflowing emotionally, to control my reaction		4.14	3.41
33. I wrote about what had happened to me, about the feelings it triggered in me, in an effort to avoid overflowing emotionally, to control my reaction		2.68	3.24
34. I accepted and endured the situation, trying to get on with normal life		6.54	2.97
*Gratitude and self-reward* (F)	**0.87**		
**28**. I treated myself to something special		4.23	2.95
**30.** I tried to think about those things that are going well for me		5.47	3.05
**31**. I tried to be grateful for the things in my life that are going well		6.16	3.41
*Spiritual activities* (F)	**0.89**		
**36.** I tried to cope spiritually, put my faith in God, or did something religious		2.23	3.20
40. I read or did something religious, of a spiritual nature.		1.73	2.78
*Reappraisal* (F)	**0.91**		
**37.** I tried to reinterpret the situation, to find a different meaning		5.02	3.09
**38.** I tried to put things in perspective		5.69	3.08
**39**. I tried to find something good in the situation.		4.95	3.35
*Social comparison* (D)	**0.69**		
**42.** I compared myself to people who are worse off		3	2.78
43. I compared myself to people who have more resources, personal resources, and done better than me, to improve the situation.		2.39	2.58
**Response modulation-** *split-half reliability- α_1_ = 81, α_2_ = 0.81, r = 0.69*			
*Inhibition/suppression* (D)	**0.80**		
10. I tried not to think about what had happened, to ignore the emotions I was feeling		2.95	2.34
**11.** I tried to not let my feelings show, to suppress any expression		3.58	2.83
12. I faked, or expressed emotions opposite to those I was feeling		2.75	2.69
*Active physiological regulation* (F)	**0.78**		
**15.** I played sports, exercised		3.02	3.04
16. I practiced relaxation, meditation		2.61	3.05
*Passive physiological regulation* (D)	**0.79**		
**17.** I slept or took a nap		3.35	3.15
**18.** I ate something to get over my bad mood		3.24	3.19
**19.** I drank coffee or caffeinated beverages		2.36	3.08
**20**. I used alcohol to get out of a bad mood		1.56	2.39
*Humor, warmth* (F)	**0.61**		
**26.** I laughed, joked around, tried to make myself or others laugh		5.03	3.07
27. I expressed myself or behaved more affectionately, sought erotic enjoyment.		2.92	2.62
*Venting*	**0.87**		
**44.** I let my feelings out by venting or expressing them		4.89	3.16
45. I made my emotion clear, verbalizing it and expressing it as strongly as I could with my face, my gestures and my way of behaving		4.16	3.01
*Confrontation* (D)	**0.75**		
46. I expressed my feelings to the person(s) responsible for the situation or tried to get them to change their minds or to improve the situation		4.42	3.17
47. I spoke sarcastically or ironically to/about the person(s) responsible for the situation		2.91	2.61
48. I showed my emotions to the person(s) responsible for the situation, behaving differently toward them		3.17	2.84
*Regulated expression* (F)	**0.64**		
49. I kept my feelings under control while it was convenient, and later, when they would not make matters worse, I expressed them		4.46	3.06
**50.** I wrote about my feelings in a diary, letter, or e-mail		2.52	3.32
51. I calmly apologized for what was done and said		3.32	2.94

*Bold items represent original version mood affect regulation- Larsen and Prizmic (2004). F, functional strategies; D, dysfunctional strategies defined by Aldao and Nolen-Hoeksema (2012), Aldao (2013)*.

#### The Emotion Regulation Questionnaire, ERQ (Gross and John, 2003; Páez et al., 2012)

The ERQ is a self-report questionnaire that measures dispositional emotional regulation, which consists of two scales corresponding to two different emotion regulation strategies: reappraisal (e.g., ‘When I want to feel more positive emotion I change what I’m thinking about’) and expressive suppression (e.g., ‘I keep my emotions to myself’). It has 10 items which are answered using a 7 point scale, from 1 (strongly disagree) to 7 (strongly agree). In this study alpha coefficients were 0.69 and 0.57, respectively. High scores indicated greater dispositional emotional regulation.

#### Positive and Negative Affect Scale, PANAS (Watson et al., 1988)

This scale contains 20 mood descriptors (e.g., active, excited, hostile, etc.) which are relatively pure markers of either high negative affect (NA) or high positive affect (PA). In the Spanish version Cronbach alpha for NA was 0.80 and 0.68 for PA (Velasco and Páez, 1996). The reliability in the present sample of the positive mood was 0.85, and for negative mood 0.89. Items are answered using a 5-point scale, from 1 (never), to 5 (always). High scores indicated greater presence of negative or positive affect.

#### Psychological Well-Being Scale, PWB (Ryff, 1989; Adapted and Validated by Díaz et al., 2006)

Ryff (1989) developed a theoretically based self-report inventory designed to measure six dimensions of psychological well-being. The six dimensions are self-acceptance, environmental mastery, purpose in life, positive relations with others, personal growth, and autonomy. Responses were recorded on a 5-point Likert scale ranging from 1 (strongly disagree) to 6 (strongly agree). The reliability in the present sample of the composite PWB score was high (α = 0.90). High scores indicated greater psychological well-being.

### Procedure

The study used a descriptive, correlational, and cross-sectional design. Participants were recruited from local universities, where research assistants administered the questionnaire during lectures. We included adults (≥18 years of age) without diagnosed personality or anxiety disorders. Instruments were administered with pencil and paper, in groups and under the supervision of research assistants. The data were collected at two moments. Participants used a code to identify themselves at both moments. First, participants were asked to select and describe an event that had caused them anger, and another sadness, choosing from a list of 12 life changing episodes. With regard to that event, they were to provide information on the type of event, its intensity, pleasant or unpleasant emotions, and the date on which it occurred. The list included negative events like problems with personal relationships, studies or work, diseases and deaths, which could be related either to anger or sadness. All participants responded with regard to each of the two emotional episodes on the same day. With regard to each of the emotional events, students were instructed to provide information on the type of event and to inform when the event had occurred. Then, they responded to MARS in relation to each specific emotional event. The data from the well-being and dispositional emotional regulation scales were collected in the class sessions 2 weeks prior to the participants completing the MARS scale. Participants (adults and students) took an average of 30 min for each practice session to complete the questionnaire. The students and adults completed the questionnaires following the same procedure. Filling in the questionnaire was volunteer. This study was carried out in accordance with the recommendations of university’s bioethical committee with written informed consent from all subjects. All subjects gave written informed consent in accordance with the Declaration of Helsinki. The protocol was approved by the Research Ethics Committee of Basque Country University.

## Results

### Descriptive Analyses

As anger evoking episodes, participants mainly selected problems related to personal relationships as triggers (66.7%). Regarding to sadness episodes, participants mainly selected experiences associated with deaths (38.6%) and personal relationships (40.5%). Both episodes had occurred over the previous 6 months. Paired *T*-tests also found no differences between sadness (intensity: *M* = 8.62, *DT* = 1.87; unpleasant/pleasant: *M* = 1.99, *DT* = 1.66) and anger (intensity: *M* = 8.45, *DT* = 7.98; unpleasant/pleasant: *M* = 2.84, *DT* = 8.26) in emotional experiences (intensity: *t* = 0.260, *p* = 0.795; unpleasant/pleasant: *t* = -1.22, *p* = 0.224). As we did not find differential impact with the aforementioned variables, we collapsed anger and sadness. Both are expressions of negative emotional experiences. We did not differentiate between them further within the analyses. Correlations between the strategies used in the two episodes are significant (ranging from 0.32 to 0.80, with mean *r* equal to 0.51). Also, there are satisfactory global reliabilities of each item across the episodes (alphas between 0.52 and 0.93) (see **Table 1**).

### Construct or Structural Validity

**FIGURE 1:**
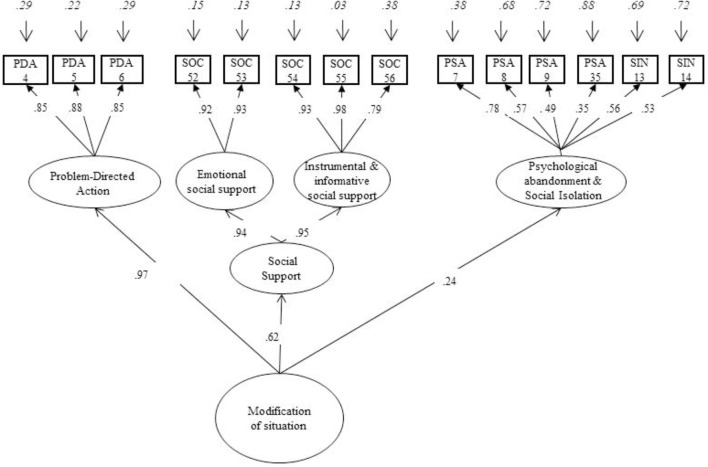
Confirmatory factor analysis (CFA). Affect regulation strategies: direct and indirect modification of situation.

**FIGURE 2:**
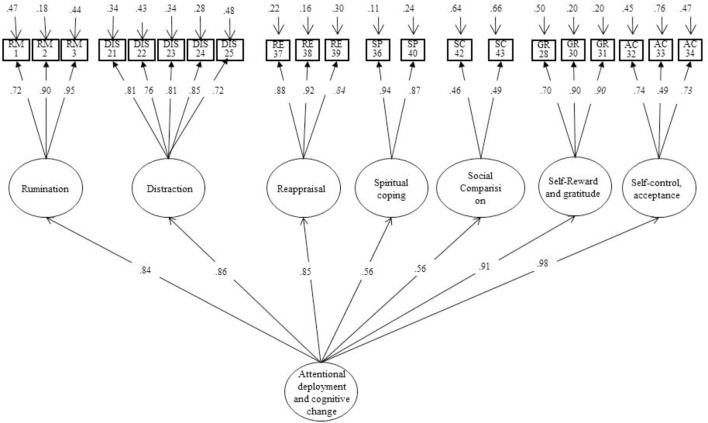
Confirmatory factor analysis (CFA). Affect regulation strategies: attentional deployment and cognitive change.

**FIGURE 3:**
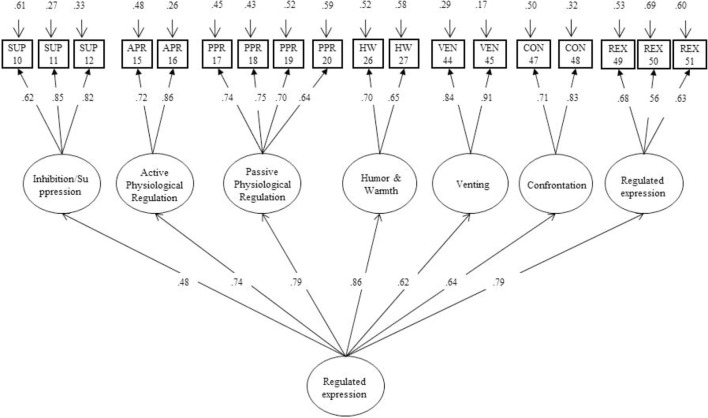
Confirmatory factor analysis (CFA). Affect regulation strategies: emotional response modulation.

Two different theoretical models are examined by a confirmatory factor analysis (CFA) with Maximum Likelihood estimation using the Mplus 7.11 software package (Muthen and Muthen, 1998–2012), for each of the sub-scales: (a) modification of the situation, (b) attention and cognitive change and (c) modulation of emotional response. Model 1, proposed by Larsen and Prizmic (2004), describes multiple forms of coping strategies regarding to the emotional process: modification of situation, attentional and cognitive change, and modulation of emotional response (Brown, 1998). Model 2 with changes on the structure of Model 1 (Páez et al., 2012), confirms and achieves a significant improvement in each of the three theoretical dimensions described by Brown (1998) and Gross (2015) (see **Figures 1**–**3**). The scaled chi-squared test is applied with the Satorra–Bentler adjustment (χ2–*SB*, Satorra and Bentler, 1994), based on the robust standard estimator. A good model fit is indicated by a Comparative Fit Index (CFI) higher than 0.90 and a Root Mean Square Error of Approximation (RMSEA) lower than 0.08 (Hu and Bentler, 1999; Kline, 2010). Finally, omega hierarchical (ω_h_) estimates the reliability of each factor with variance from the general factor removed. There are no absolute standards for evaluating the magnitude of ω or ω_h_, but it has been tentatively suggested that values near 0.75 might be preferred, and values greater than 0.50 might be a minimum (Reise et al., 2013) (see instruments).

#### Modification of Situation

The original one-factor structure composed by eight items finds to be a poor fit (Model 1) (Larsen and Prizmic, 2004): χ^2^(20, *N* = 264) = 260, *p* < 0.0001; CFI = 0.68; TLI = 0.56; RMSEA = 0.21 (95% CI [0.19, 23]), ACI = 10226.005 (see **Table 1**).

The model 2 (see **Figure 1**) proposes by Páez et al. (2012), includes 14 items and 4 first-order factors (problem-directed action, emotional social support, instrumental and informative social support, and psychological abandonment and social isolation) and one second-order factor (social support). The results suggest some modifications to the theoretical model (Model 1). First, altruism (item 41) was included in the social support dimensions, a form of reciprocal social support, but did not show a satisfactory correlation (increasing the error variance) and was excluded. Second, psychological abandonment and social isolation correlate strongly [*r*_(252)_ = 0.98, *p* = 0.0001] as well as instrumental/informative and emotional social support [*r*_(252)_ = 0.89, *p* = 0.0001]. The correlations indicate that psychological abandonment and social isolation may be measuring the same construct and thus, are not different dimensions. In addition, modification indices include item 54 (‘I talked to someone in order to resolve the situation or to improve the situation that triggered my mood’) as a form of informational social support. Finally, the fit indices are better if social support is considered as a second order factor describing the emotional, informative social and instrumental support, rather than being treated as a single factor. The data confirm that all these dimensions represent the latent factor of modification of situation: χ^2^(72, *N* = 264) = 183, *p* < 0.0001; CFI = 0.95; TLI = 0.94; RMSEA = 0.07 (95% CI [0.06, 0.09]), ACI = 16419.55. Model modification indices shows that none of the items should be included in a different factor with respect to conceptual dimensions. The indices of fit are acceptable and the change in the chi-squared value is significant in comparison with model 1 [Δχ^2^_(27)_ = 65.16, *p* < 0.001] (Hu and Bentler, 1999) (**Figure 1**).

#### Attentional Deployment and Cognitive Change

Firstly, the 1-factor, 15-item model 1 shows that, the goodness of fit indices are not adequate: χ^2^(119, *N* = 264) = 694.791, *p* < 0.0001; CFI = 0.81; TLI = 0.78; RMSEA = 0.14 (95% CI [0.13, 0.15]), ACI = 20218.193 (see **Table 1**).

The second model analyses a structure with 7 dimensions and 21 items (Páez et al., 2012). The CFA shows that the model fit improves with the exclusion of the wishful thinking item (29. ‘I daydreamed about the time when I will feel better than today’): χ^2^(182, *N* = 264) = 458.64, *p* < 0.001; CFI = 0.93; TLI = 0.92; RMSEA = 0.07 (90% CI [0.07, 0.08]), ACI = 24423.50 (see **Figure 2**). The change in chi-squared between models 1 and 2 is significant and improves the model’s fit indicators [Δχ^2^_(119)_ = 207.99, *p* < 0.001] (**Figure 2**).

#### Emotional Response Modulation

The original one-dimensional structure (Model 1) includes nine items. The Model 1 fit indices are acceptable: χ^2^(27, *N* = 264) = 81.689, *p* < 0.0001; CFI = 0.91; TLI = 0.87; RMSEA = 0.08 (95% CI [0.06, 0.11]) (see **Table 1**).

In addition, MARS model 2 includes 18 items and seven dimensions in the emotional response modulation facet. Item 46 is eliminated due to it increases the error variance (RMSA = 0.09) and decreases the model fit (CFI = 0.87, TLI = 0.84). The final CFA shows a good fit with the data: χ^2^(127, *N* = 264) = 285.80, *p* < 0.0001; CFI = 0.91; TLI = 0.90; RMSEA = 0.07 (95% CI [0.06, 0.08]). Model modification indices show that none of the items should be included in a different dimension with respect to the conceptual factor (see **Figure 3**). Furthermore, the change in the chi-squared value is significant in comparison with model 1 [Δχ^2^_(188)_ = 400.26, *p* < 0.001] (**Figure 3**).

### Convergent Validity

To obtain evidence of the convergent validity of the instrument, Pearson correlations were calculated between the scores of MARS dimensions and ERQ (reappraisal and suppression) as dispositional indices of affect regulation (see **Table 2**). The bivariate analyses confirm that reappraisal was associated positively with all forms of adaptive regulation, but also with rumination and passive physiological regulation. Suppression was associated with psychological abandonment and social isolation, suppression, low social support and venting.

**Table 2:** Correlations between MARS and ERQ, PANAS and PWB Ryff scales.

	ERQ		PANAS		PWB Ryff
	Reappraisal	Suppression	Positive	Negative	
Problem-directed action	0.24***	-0.09	0.22***	0.18**	0.21***
Withdrawal and social isolation	0.03	0.26***	0.02	0.33***	-0.17***
Social support	0.26***	-0.33***	0.27***	0.11	0.26***
Distraction	0.31***	-0.03	0.17**	0.24***	0.14*
Acceptance and self-control	0.32***	-0.02	0.26***	0.15*	0.25***
Gratitude and self-reward	0.36***	-0.01	0.22***	0.11	0.19***
Spiritual activities	0.20***	0.03	0.02	0.10	0.12
Rumination	0.22***	-0.02	0.19**	0.28***	0.13
Reappraisal	0.36***	-0.07	0.20***	0.06	0.25***
Social comparison	0.11	-0.05	-0.044	0.01	-0.05
Inhibition and suppression	0.05	0.43***	-0.64	0.33***	-0.18**
Active physiological regulation	0.25***	0.06	0.16*	0.16*	0.18***
Passive physiological regulation	0.17***	-0.02	0.01	0.01	0.03
Humor, Warm	0.25***	0.01	0.17**	0.16*	0.13
Venting	0.10	-0.36***	0.26***	0.05	0.28***
Confrontation	0.11	0.06	0.06	0.08	0.07
Regulated expression	0.29***	0.1	0.19***	0.09	0.20***

*^∗∗∗^*p* < 0.001, ^∗∗^*p* < 0.01, and ^∗^*p* < 0.05*.

In order to contrast the association between coping and affect regulation strategies with hedonic adaptive goals, factor scores are correlated with PANAS positive and negative affect scores (see **Table 2**). Maladaptive forms of regulation like withdrawal and social isolation, and suppression are associated with higher negative affect. Problem-directed action, distraction, acceptance, rumination, active physiological regulation and use of humor are positively and significantly related to both, positive and negative affect. Social support, gratitude and self-reward, reappraisal as well as venting and regulated expression are significantly associated with positive affect and not significantly with negative effect.

With the goal of examining the association between previously described forms of regulation and instrumental and social-adaptive goal factors, scores were correlated with Ryff’s PWB scores (see **Table 2**). As expected, problem-directed action and planning, seeking social support, attentional deployment through distraction, acceptance and gratitude/self-reward, cognitive change by reappraisal, response modulation by active physiological regulation, venting and regulated expression correlate positively with psychological well-being. Withdrawal, social isolation and suppression were associated with low psychological well-being.

### Discriminant Validity Between Groups of High and Low Hedonic and Psychological Well-Being

Discriminant function analysis (DFA) and analysis of variance (ANOVA) are used to examine the differences in affect regulation between groups. Hence, the overall analyses sought to answer the questions of how each strategy: (a) contributes to predicting group assignment and (b) represents significant mean differences among groups.

To explore a combination of well-being, we create four groups based on the median of PWB and PANAS (high PWB and PANAS; high PWB and low PANAS; low PWB and high PANAS, and low PWB and PANAS). Discriminant analysis between these groups found one statistically significant canonical discriminant function (see **Table 3**) which explains 68.2% of the variance in the use of affect regulation strategies differentiating between groups [Wilks’ λ = 0.61, χ^2^_(51)_ = 104.65, *p* = 0.0001].

**Table 3:** Items discriminating between Flourishing group, low PANAS and high PWB, high PANAS and low PWB, and languishing group.

Item	PWB Ryff ^∗^PNAS											
	High PWB^∗^PANAS (*n* = 70)		High PWB^∗^low PANAS (*n* = 74)		Low PWB^∗^ high PANAS (*n* = 50)		Low PWB^∗^PANAS (*n* = 70)		*Wilks’* λ	*F*	Function Loading	η^2^
	*M*	*DT*	*M*	*DT*	*M*	*DT*	*M*	*DT*				
**Modification of situation**						
Problem-directed action	3.72	1.43	4.18^a^	1.18	3.47b	1.25	3.68^b^	1.37	0.95	3.36**	0.07	2.35
Social support	3.52	1.70	3.90^a^	1.59	3.28	1.70	3.34^b^	1.55	0.96	2.98*	-0.36	2.06
Withdrawal and social Isolation	1.72^a^	0.96	1.93	0.99	2.30	0.86	2.74^b^	1.15	0.93	4.23**	-1.02	2.97
**Attentional deployment and cognitive change**						
Distraction	3.58	1.25	3.77	0.94	3.70	0.92	3.67	1.14	0.97	2.41	-0.11	–
Acceptance and self-control	2.86	1.08	3.29^a^	1.34	2.50^b^	1.01	2.53^b^	1.04	0.91	6.52***	1.03	4.05
Gratitude and self-reward	3.45^a^	1.30	3.55^b^	1.19	3.28	1.29	2.99^b^	1.36	0.95	3.53*	0.38	2.41
Spiritual activities	1.04	1.58	1.55^a^	1.91	0.69^b^	1.04	0.85^b^	1.38	0.95	3.67*	0.51	2.42
Rumination	3.60^b^	1.21	4.29^a^	0.91	3.76^b^	0.93	3.99^b^	1.08	0.95	4.23**	0.09	2.97
Reappraisal	3.39	1.52	3.73^a^	1.51	3.20^b^	1.11	2.94^b^	1.42	0.93	5.88**	-0.11	4.03
Social comparison	1.62	1.37	1.64	1.50	1.88	1.54	1.95	1.46	0.99	0.21	-0.05	–
**Response modulation**						
Inhibition and suppression	1.59	1.28	1.77^a^	1.11	2.02	1.29	2.50^b^	1.51	0.96	2.78*	-0.14	1.91
Active physiological regulation	1.82	1.43	1.92	1.80	1.69	1.40	1.46	1.46	0.97	1.72	0.22	–
Passive physiological regulation	1.51	1.27	1.78	1.58	1.55	1.33	1.93	1.16	0.98	1.01	-0.33	1.91
Humor, warmth	2.49	1.31	2.65	1.26	2.73	1.25	2.46	1.42	0.96	2.78*	-0.06	–
Venting	3.21	1.63	3.15	1.51	2.74	1.58	2.89	1.51	0.97	2.15	0.11	–
Confrontation	1.96	1.75	1.87	1.14	2.19	1.64	2.39	1.88	0.99	0.02	0.21	–
Regulated expression	2.2	1.43	2.44^a^	1.32	1.87^b^	1.09	2.11^b^	1.26	0.96	2.69*	-0.12	1.90

*17 total affect regulation strategies; Likert-type response scale ranged from 0 (never) to 6 (nearly always). Different sub-indices indicate significant differences between groups (Bonferroni correction). ^∗^p < 0.05, ^∗∗^p < 0.01, ^∗∗∗^p < 0.001.*

Specific items comprising Function 1 can be found in **Table 3** along with one-way ANOVAs, significant *post hoc* differences, group mean, and standard deviations. Eta square effect size shows that type of wellbeing explains between 1.9 and 4% of variance. Significant group differences are found between the high PWB-low PANAS and low PWB- low PANAS^,^ group in problem-directed action, social support, acceptance and self-control, gratitude, spiritual activities, rumination, reappraisal and regulated expression. Besides, languishing individuals report using more inhibition and suppression strategies than the individuals with high PWB and low PANAS. In terms of the *post hoc* analyses, participants with high PWB and PANAS report higher gratitude and lower withdrawal and social isolation than the languishing group. Also, participants with high PWB and PANAS report lower rumination than the high PWB-low PANAS. Furthermore, high PWB-low PANAS present higher scores than low PWB-high PANAS in problem-directed action, acceptance, spiritual activities, rumination, reappraisal and regulated expression.

## Discussion and Conclusion

This paper empirically evaluates Gross’s Process Model of Emotion Regulation and validates an expanded version of the MARS in negative emotional episodes. Globally, our results confirm the structural validity of dimensions of regulations and types of strategies. One of the most important findings of this study is that various forms of affect regulation show a reliable structure in different aspects or phases of affect regulation. Also, it provides an instrument that enables reliable diagnoses of functional self-regulation. Confirmatory factor analyses support the structure of expanded MARS (Páez et al., 2012). Consistent with previous research, the results of these analyses show a satisfactory fit with the three affect regulation systems: direct and indirect modification of situation through asking for social support, deployment of attention and cognitive change, and emotional response modulation. Furthermore, the expanded version of the scale implies an improvement regarding the original structure proposed by Larsen and Prizmic (2004) (Model 1).

First, the data show that both emotional and cognitive instrumental social support load together in the second factor, differentiating between instrumental/informative and emotional social support. Unfortunately, and at odds with the conception that receiving and giving social support are integrated in a common process, coping by helping others or altruism did not fit adequately. Only one item was used which did not allow testing the existence of a separate dimension. Asking for social support during distress does not necessarily imply a similar orientation toward giving social support – the latter is probably associated with high self-efficacy and prosocial values. Furthermore, CFA also suggests that withdrawal and social isolation should be considered together, forming an avoidance dimension strategy that is clearly dysfunctional. Both of them refer to taking actions that directly alter a situation in order to change its emotional impact, and although these situation-modifying behaviors lead to short-term relief, they prevent full exposure to the feared situations, preventing longer term benefits of exposure (Gross, 2015). Second, the strategies of attention deployment and cognitive change show a good fit with the data. The second family includes 21 items and 7 dimensions referring to distraction, acceptance and self-control, gratitude and self-reward, spiritual activities, rumination, reappraisal and social comparison. In fact, these strategies suppose a positive revaluation of behaviors or negative emotional situations (Larsen and Prizmic, 2008). A previous research has also found that distraction and reappraisal are strongly related in affect regulation. Even if items try to represent mainly attentional effort versus cognitive processing, in other CFAs attentional items load in reappraisal and vice versa (da Costa et al., 2014), probably because attention and thinking are intrinsically connected processes.

Finally, the items related to dimensions of emotional response modulation show good fit with the data. The final model includes 18 items and 7 dimensions; suppression, active and passive psychological regulation, humor and warm, venting, confrontation and regulated expression. All of these coping strategies involve attempts to directly influence emotional response system (Koval et al., 2014). In sum, expanded MARS (Páez et al., 2012) could be considered relatively satisfactory due to none of the items in each dimension are included in different family according to the conceptually postulated models.

Convergent validity of the scales is also confirmed. Results suggest that there is a congruent relationship between dispositional indicators of emotional regulation and using functional and dysfunctional strategies in episodes of anger and sadness. Reappraisal is associated with high use of problem-directed action and social support, and attentional deployment and cognitive change strategies (except social comparison); moreover, it is associated with high active and passive psychological regulation, humor, warmth and regulated expression. Suppression is associated not only with withdrawal, social isolation and inhibition, but also with low social support and venting (Naragon-Gainey et al., 2017; Schäfer et al., 2017).

Results support a congruent relationship between the use of functional and dysfunctional strategies in episodes of anger and sadness and indicators of hedonic and psychological well-being (Seligowski et al., 2015). Forms of affect regulation were also related to well-being (Koval et al., 2014; Visted et al., 2018). Results confirm that withdrawal and social isolation, and suppression are associated with negative high self-reported affect over the past month, and to low psychological well-being. These forms of regulation are detrimental for hedonic, instrumental and social goals (English et al., 2017).

Problem-directed action, distraction, acceptance and self-control, rumination, active physiological regulation, use of humor and affection were related to positive and negative affect. Most of these forms are a response to emotional stress and are associated with negative affect as a coping response. Social support, gratitude and self-reward, reappraisal, and regulated expression are only associated with positive affect and not to negative affect (Brockman et al., 2017). This means that these forms could be conceived as being based on dispositional positive affect or as improving positive affect when coping with negative events. However, venting and rumination could be understood as fueled by negative affect (Aldao et al., 2010; Renna et al., 2018).

As expected, psychological well-being was associated with modifying the situation through problem-directed action and seeking social support. PWB was also associated with attentional deployment through distraction, acceptance/self-control and gratitude/self-reward and to cognitive change by reappraisal (English et al., 2017). Finally, PWB correlates with response modulation by active physiological regulation, and regulated expression. In other words, results also confirm that these forms of regulation were associated with perceived control of the situation, high self-esteem and positive relationships with others as measured by Ryff’s PWB scale. Because all of them are also related to positive affect, these forms appear to be connected with improved hedonic, instrumental and social-adaptive goals.

Venting, thought as to be a negative form of regulation, was associated with psychological well-being, positive affect and low suppression. Results suggest that intense emotional expression is not necessarily dysfunctional. This is congruent with a non-significant positive association between venting and affect balance (Páez and Da Costa, 2014). Recent studies conclude that venting and confrontation could be adaptive if they are associated with regulated expression, probably because both are related to assertiveness and to elicit and receive social support (Campos J.J. et al., 2004; da Costa et al., 2014). Rumination has an ambivalent profile. It was associated with positive affect and high reappraisal, suggesting that is linked with positive forms of emotional processing. However, it is at the same time related to negative PANAS, confirming the association between repetitive thinking and anxiety (Aldao et al., 2010; Jamieson et al., 2013).

Discriminant analysis shows an interesting result. The forms of affect regulation that most significantly characterize languishing participants with low PWB and hedonic well-being were high psychological abandonment, social isolation, low social support and high inhibition/suppression. This is an important finding, because it reaffirms the central role of social relations and low helplessness for well-being. Furthermore, this group usually used gratitude and self-reward less than other groups (except low PWB, high PANAS). Flourishing subjects (high PWB and PANAS) report highest gratitude and low social isolation and psychological abandonment. Attentional deployment positively oriented and absence of social and behavioral avoidance, appeared as the mark of subjects with the most positive well-being profile when regulating negative events. The most adaptive profile was reported by participants with high PWB and low positive affect, which reported highest modification of situation (problem-directed action and social support), acceptance and self-control, gratitude and self-reward, spiritual activities, rumination, reappraisal and regulated expression, and lower inhibition. The fact that an adaptive profile was showed by participants with high psychological well-being (Korpela et al., 2018), even if they reported low positive and high negative affect, suggests the relevance of eudaimonic over hedonic well-being. Results can be interpreted also in the sense that participants with high psychological well-being but with an affect balance below the median cope with this unpleasant hedonic state by deploying strong affect regulation. However, because of the correlational character of this study it is difficult to clarify whether these associations show a form of coping with negative affect or if they are elicited by high negative affect. In addition, PANAS was answered with regard to the previous months and globally negative episodes were lived 6 months prior the test whereas stress episodes influence the last 3–6 months (Schimmack, 2008; Gross, 2015). It is possible that these people had salient coping efforts in the aftermath of recent negative events.

In conclusion, the results show a significant, medium-low correlation between forms of regulation with hedonic and psychological well-being. At the same time, the study replicates the association of adaptive regulation with well-being, and supports the structural validity of the MARS scale, as well as convergent validity with ERQ’s suppression and reappraisal. There is an association between dysfunctional emotional regulation (high coping by withdrawal and social isolation and suppression) and low hedonic and psychological well-being. In addition, the measure was found to be reliable and valid, with the construct validity supported by associations with conceptually relevant constructs accessed via self-report measures. Information regarding the specific difficulties that participants experience in response to particular types of cues or stressors could also be used to enhance the targeted and tailored nature of interventions in other contexts (i.e., clinical). The results of this study are useful for promoting emotional capacities for coping more effectively with negative situations in educational contexts. These results provide guidance on how to implement intervention programs, aimed at enhancing well-being and reducing psychosocial maladjustment in negative situations. Adaptive strategies are forms of regulation that should be reinforced as ways to improve affective well-being. To help people overcome a state of low emotional and psychological well-being, it is essential to reduce their tendencies toward psychological abandonment, social isolation, low social support and high inhibition/suppression. This means that increasing successful social integration and self-efficacy is essential for well-being. In this line of reasoning, teaching students to use positive reappraisal and acceptance can foster healthy skills to help them face adverse circumstances in the future.

In contrast, this study has clear limitations: the conclusions are based on the correlational analysis of self-reports. The sample size may have been too small, and further larger studies are required to confirm these results. More research is needed to fully understand the complex relationships among these constructs. The cross-sectional design of the study limits any conclusion about causality and direction of relationships. Measuring affect regulation with retrospective self-reports is another limitation because self-reported emotion regulation is not the same as actual measurement of affect regulation on-line. On-line experimental studies, as well as observational longitudinal studies, are needed to expand our understanding of affect regulation. Nevertheless, our results are globally congruent with the findings of both experimental (Augustine and Hemenover, 2008) and longitudinal studies (Nezlek and Kuppens, 2008), as well as with previous meta-analyses (Aldao et al., 2010; Webb et al., 2012). As such, future research examining the factor structure and psychometric properties of the MARS in response to a variety of naturalistic and/or laboratory-based stressors is needed. Also, the examination of the psychometric properties of the MARS in relevant populations that are characterized by higher levels of emotion dysregulation would be a useful direction for future research.

## Author Contributions

AP-M data analysis and wrote the paper. DP and SU-L wrote the paper. SDC-D collected the data.

## Conflict of Interest Statement

The authors declare that the research was conducted in the absence of any commercial or financial relationships that could be construed as a potential conflict of interest.
